# On the Use of Coffee in Infantile Cholera

**Published:** 1849-12

**Authors:** 


*On the Use of Coffee in Infantile Cholera.* By Dr. Pickford.—Dr. Pickford states, that from the great importance which now attaches to the treatment of cholera, he feels it to be incumbent upon him to impart to others the experience which recent opportunities have afforded him of the effects of coffee in the cholera of infants.

In the case of an infant at the breast, to which he was called late, to whom the usual remedies had been admin istered unavailingly for four days, the exhibition of coffee was attended with complete success. The incessant vomiting and purging had produced extreme emaciation: the abdomen was distended; the pulse was frequent and small; there was great restlessness, and sleeping with the eyes half opened; convulsive motions of the eyes when awake. Carbonate of ammonia, with nourishing diet, and external stimulants, having been fruitlessly exhibited Dr. Pickford determined to have recourse to coffee, which he knew to have been recommended as a stimulating tonic, by Dr. Dewees. He began with a small dose, a scruple, infused in two ounces of water, with one ounce of syrup, giving a large spoonful every hour. The effect was surprising; the vomiting was arrested; the evacuations became more consistent, improved in color, and less frequent. The amendment progressed so rapidly, that by the tenth day the child was discharged as cured.

The effects were equally good in a little girl, fourteen weeks old, in whom the vomiting was not so severe, but the diarrhea was quite as copious. In this case also coffee was given, after other means had been tried, and the patient greatly reduced. Dr. Pickford has since used this remedy in nine children of different ages, from four weeks to two years and a half. The doses have varied from half a scruple to two scruples daily. He has also administered it to children laboring under premonitory symptoms, especially where the evacuations have been very light colored. In some cases a single dose of calomel has preceded its employment. The effect was always favorable, except in one case to which he was called too late, when the child was already sinking. He has not had any occasion to try the value of coffee in the diarrhea of adults, having found calomel and opium of sufficient efficacy.

The benefit of coffee, especially in bilious diarrhea, has been extolled by Lauzow and Chultze (Richter's Arzneimittellehre, vol. 1). West, in 1813, found a combination of coffee and opium very useful in the epidemic of that year. Coffee has long been employed by the common people as a remedy (in Germany, we suppose), after excessive indulgence in spirit drinking. It is known to have the property of promoting digestion, and the action of the bowels. The purgative action of burnt coffee is attributed by Dr. Pickford to its tonic exciting properties. Like some other substances, in small doses it is capable of restraining diarrhea, while in large doses it acts as a cathartic. The physiological explanation of this opposite effect of the same remedy is probably to be found in the condition of the motor nerves, which, being weakened, are by its moderate stimulus restored to their normal state of excitement, and thereby diarrhea depending on their paralysis is cured. In this way, also, is explained its aperient action in larger doses on adults, by its overstimulating these nerves, and so promoting increased movements of the intestines. This simple domestic remedy is so readily available that its efficacy deserves to be tried, and, if confirmed, made more extensively known. Our own experience of its utility in a case of vomiting and purging which has recently presented itself to us fully bears out the author's encomiums. It is not, however, safe to draw conclusions from a single instance.—*Med. Gaz.*

